# The pattern of substance use disorder in the United Arab Emirates in 2015: results of a National Rehabilitation Centre cohort study

**DOI:** 10.1186/s13011-016-0062-5

**Published:** 2016-05-13

**Authors:** Hiba Alblooshi, Gary K. Hulse, Ahmed El Kashef, Hanan Al Hashmi, Mansour Shawky, Hamad Al Ghaferi, Habiba Al Safar, Guan K. Tay

**Affiliations:** Centre for Forensic Science, University of Western Australia, Crawley, 6009 WA Australia; School of Psychiatry and Clinical Neurosciences, University of Western Australia, Crawley, WA 6009 Australia; United Arab Emirates National Rehabilitation Center, Abu Dhabi, United Arab Emirates; Center for Biotechnology, Khalifa University of Science, Technology and Research, Abu Dhabi, United Arab Emirates; Faculty of Biomedical Engineering, Khalifa University of Science, Technology and Research, Abu Dhabi, United Arab Emirates

**Keywords:** Substance-related disorders, Opioid-related disorders, Alcohol drinking, Prescription medication use, United Arab Emirates

## Abstract

**Background:**

Substance use disorder (SUD) is a global problem with no boundaries, which also afflicts individuals from countries of the Arabian Peninsula. Data from this region is limited. In an effort to develop targeted prevention and intervention initiatives in the United Arab Emirates (UAE), it was necessary to identify the nature of substance use by describing the characteristics of those using different substances. Consequently, this study in the UAE was conceived to describe the pattern of SUD in a first-ever cohort that was systematically recruited from the country’s National Rehabilitation Centre (NRC) in Abu Dhabi.

**Methods:**

Two hundred and fifty male patients were recruited from the NRC. Information on substance use was collected using a questionnaire that was completed at an interview with patients who consented to participate. The questionnaire was based on information that the study was designed to capture. It was reviewed by members of institutional ethics committees and approved prior to use. Two hundred and fifty male subjects from the Emirates Family Registry (EFR) were used as a comparison group.

**Results:**

In the cohort studied, SUD correlated with smoking and marital status. Poly-substance users formed the majority of the cohort (84.4 %) with various combinations of substances identified across different age groups. Opioid and alcohol were the most common substances used. The use of pharmaceutical opioids, primarily Tramadol (67.2 % of opioid users), was higher among the youngest age group studied (<30 years old), while older opioid users (≥30 years old) commonly used illicit opioids (Heroin). The use of prescribed medication for non-medical use also included Pregabalin (mean of 8.3 capsules ± 0.5 per day), Procyclidin (6.1 tablets + 0.6 per day) and Carisoprodol (4.2 tablets ± 0.4 per day) and was again highest in the age group below 30 years.

**Conclusion:**

This 2015 study highlights the importance of examining the pattern of poly-substance use in a population in order to develop targeted prevention programs to arrest the prevailing trends. It has drawn attention to the rise in use of prescription medication in the UAE, in particular among younger patients (<30 years), and continuing use of illicit opioid amongst males above 30 years. Specific prevention and intervention strategies, targeting differences between these distinct demographic profiles will capture a large subset of sufferers.

## Background

The fifth edition of the Diagnostic and Statistical Manual of Mental Disorder (DSM-5) combines the classification of substance abuse and dependence in the disease referred to as substance use disorder (SUD) [1]. The criteria for diagnosis were increased by two or more points out of 11. Poly-substance dependence has been removed from the latest edition due to the lack of specific substance preference and failure to meet the dependence criteria collectively across substances [2]. These diagnostic challenges has necessitated that the DSM-5 classification for substance use disorder be modified such that each substance is classified as a separate use disorder [3].

Substance use is occurring in Arab countries including the United Arab Emirates (UAE) [4]. Despite strong religious censure, cultural disapproval and the illegal nature of drug trafficking, residents of the UAE are not insulated from the global epidemic that is substance use disorder. The increase in the prevalence of the disease can be attributed to various factors including the geographical location of the UAE. The country location borders southwest Asia, positioning it on the route between the countries that cultivate and produce illicit substances and the worldwide consumer market [5]. Based on the 2014 world drug report, the UAE is a primary transit country for air-trafficking of illicit substances, playing a key role in the global distribution of narcotics [6]. The rapid population growth and social drift are other contributing factors, primarily affecting the younger population [5]. The disease in the UAE and in patients of Arabian ancestry have yet to be studied extensively, and details are required to ensure that appropriate preventative measures can effectively target at-risk groups. In 2009, three studies [7–9] based on data retrieved for patients that were admitted to psychiatric wards in hospitals and clinics across the Arabian Peninsula were reviewed [5]. The review examined specific socio-demographic variables and discussed the most common substances of use, being alcohol, heroin and cannabis (hashish). Data from hospitals only provide insights into end-stages of the disease and more information is required, prompting this specific study.

The National Rehabilitation Centre (NRC) in Abu Dhabi is the primary rehabilitation facility that recruits UAE nationals from all seven emirates/principalities. Information collected from this point of care provides an objective perspective of the pattern of use in the community. The NRC provides pharmacotherapy, rehabilitation programs and long-term treatment management plans that are customised to the needs of patients. The centre also plays a role in disseminating educational material relating to a range of community oriented preventative programs. These programs target family units, children at schools, the workplace and are promoted at general public spaces in order to instill an understanding in the community of the dangers of substance use disorder as well as to introduce the available support and care in the NRC. In addition, a study was conducted by the NRC in 2013 to gauge the attitude of the community toward substance use disorder via a targeted questionnaire during a concerted prevention and awareness campaign in the city of Abu Dhabi [10]. Religious values and socio-demographic factors were identified as the two most common factors that contribute to the disorder. The majority (94 %) of the participants preferred a rehabilitation strategy when apprehended with illicit substances rather than imprisonment. A retrospective study conducted by the NRC reported that alcohol (41.3 %) followed by heroin (16.3 %) were the most common substances used among patients [11]. The use of prescription medication and other psychoactive substances was observed among poly-substance users. However, specific details of the substances used and the co-use of prescription medication was not investigated. No previous study has examined the combination of the substances used among poly-substance users in the region that is the Arabian Peninsula. Anecdotal observations at the NRC suggested that there were a large number of poly-substance users, necessitating this study to document the pattern of simultaneous substance use across patients.

This report is a natural extension of a previous NRC study conducted by Elkashef et al. (2013) and adds knowledge pertaining to the patterns that are emerging for substance use disorder among a contemporary cohort of patients in the UAE population [11]. It also describes the combinations of substances that are common among the various age groups presenting at the NRC. In addition, it describes the types of prescription and pharmaceutical substances that are commonly used. This knowledge is intended to facilitate the development of effective targeted prevention and intervention programs. This study also provides an updated epidemiological observation of a case–control cohort that was recruited for a Genome Wide Association Study (GWAS).

## Methods

### Subjects

The majority of individuals admitted to the NRC in Abu Dhabi, UAE are male patients; hence it was only feasible to study one gender. During the course of study, a total of 266 were interviewed where 251 UAE nationals provided informed consent and participated in the study. From the participants one withdrawal case was recorded. All participants were clinically diagnosed with substance use disorder based on the DSM-5 criteria. In addition, each substance of use was recorded for patients who were poly-substance users.

A group of individuals with no prior history of substance use disorder were recruited from Emirates Family Registry (EFR) for comparison group in this study. The EFR was established for research purposes in 2009 [12] and contains information on participants who were recruited from across the UAE. For this study, 250 were retrieved, and 239 were selected to ensure that the range of ages, the spread of the age groups and the means were similar to that of the study cohort.

### Ethics approval for involving human subjects in this study

The study was conducted in accordance with the ethical guidelines of World Medical Association of Helsinki [13]. Approval to conduct this research involving humans was obtained from the NRC in Abu Dhabi, UAE. Co-approval was received from the Human Research Ethics Committee of the University of Western Australia (RA/4/1/6715).

### Questionnaire and collected data

A questionnaire was completed with each participant at the NRC during a one-to-one interview session. Patients who participated in this study consented to: (1) complete the interview to provide biophysical and clinical data, (2) undergo a treatment plan outlined by the NRC, (3) provide bio-specimens for genetic studies, and (4) allow de-identified data to be used for research purposes.

Demographic data consisted of information such as date of birth, biophysical, ethnicity, lifestyle, marital status, employment status, and education level. The clinical data collected detailed substances of use, substance use history, age of initiation, symptoms experienced during use, overdose incidents and pharmacotherapy treatment received. In addition, prevailing medical conditions (diabetes, high blood pressure) were collected.

The height and weight of patients were measured to allow calculation of Body Mass Index (BMI). Blood pressure was determined at time of presentation. A saliva sample was collected using DNA Oragene saliva kit (DNA Genotek, Ottawa, Ontario, Canada) from each patient. In addition, a urine sample was collected at each follow-up visit for toxicology screening.

### Statistical analysis

The means, standard deviations and percentages of each trait were calculated using the STATA statistical software (College Station, TX, USA). *P-values* calculated using the chi-square test; with values that were less than 0.05 considered to be significant. The G*power by Faul et al. (2007) [14], was used to calculate the sample power during the design phase of this study.

## Results

Details of the study cohort comprising 250 patients (mean age: 29.6 years, minimum: 18 years, maximum: 62 years) and 239 participants (32.8 years, minimum: 18 years, maximum: 68 years) that were used as a comparison group are summarised in Table 1. Significant factors associated with substance use were smoking and marital status. Most of the patients (95.6 %) were current smokers at time of interview in contrast to only 19.7 % of the comparison group (*P-value* < 0.001). Over half the patients (58.4 %) were never married compared to 48.9 % in the comparison group (*P-value* < 0.001). Various other factors were recorded in the cohort as not significant when the two groups were compared. Seventy percent of the patients had no medical history that require medications with 72.8 % in the comparison group (*P-value* > 0.05). The majority of the patients cohort fell within the normal BMI range (18.5–24.9). The comparison group was more likely to partake in exercise compared to the patient group. More than half the patients had a family history of substance use disorder (56.8 %). Among these patients, 35.2 % were first-degree family members only (i.e., parents, siblings) and 22.4 % had both first and second or third degree relatives. Comparisons were not possible for family history, employment status and education level, as information was only available for patients with substance use disorder, and not for the comparison group.

**Table 1:** The demographic characteristics of 250 substance use patients were compared to and 239 non-users comparison in this study

Category	Description	Case n (%)	Comparison n (%)
Age groups	Below 20	12 (4.8)	22 (9.2)
	20–29	144 (57.6)	130 (54.4)
	30–39	54 (21.6)	14 (5.9)
	40–49	32 (12.8)	23 (9.6)
	Over 50	8 (3.2)	50 (20.9)
Body Mass Index (BMI)	Underweight (<18.5)	13 (5.2)	17 (7.1)
	Normal range (18.5–24.9)	93 (37.2)	79 (33.0)
	Overweight (25.0–29-9)	75 (30.0)	79 (33.0)
	Obese (≥30.0)	69 (27.6)	64 (26.8)
Medical condition^a^	No	177 (70.8)	174 (72.8)
	Yes	73 (29.2)	61 (25.5)
Smoking	Current-Smoker	239 (95.6)	49 (20.5)
	Ex-Smoker	11 (4.4)	25 (10.5)
	Never Smoked	0 (0.0)	164 (68.6)
Exercise^b^	No	122 (48.8)	84 (35.1)
	Yes	122 (48.8)	149 (62.3)
Ethnicity	Ajam	17 (6.8)	45 (18.8)
	Arab	156 (62.6)	164 (68.6)
	Bloush	18 (7.2)	10 (4.2)
	Mixed^c^	48 (19.3)	2 (0.8)
	None of the above	10 (4.0)	18 (7.5)
Family History	No	99 (39.8)	N/A^d^
	Yes	142 (56.8)	
	Not sure	9 (3.6)	
Marital status^b^	Single	146 (58.4)	117 (48.9)
	Married	89 (35.6)	81 (33.9)
	Divorced	14 (5.6)	1 (0.4)
	Widowed	1 (0.4)	0 (0.0)
Employment	Employed	82 (32.8)	N/A^d^
	Employed/Student	2 (0.8)	
	Student	31 (12.4)	
	Unemployed	116 (46.4)	
	Others	19 (7.6)	
Education level completed	Master degree	2 (0.8)	N/A^d^
	Bachelor degree	11 (4.42)	
	Diploma	30 (12.5)	
	High School (A-level)	71 (28.5)	
	Pre-high school	135 (54.2)	

^a^Medical condition means: disease or illness diagnosed other than dependency

^b^Some missing data (do not add up to the total)

^c^Mixed: Parents from different ethnic groups

^d^N/A: Not available

**Fig. 1:**
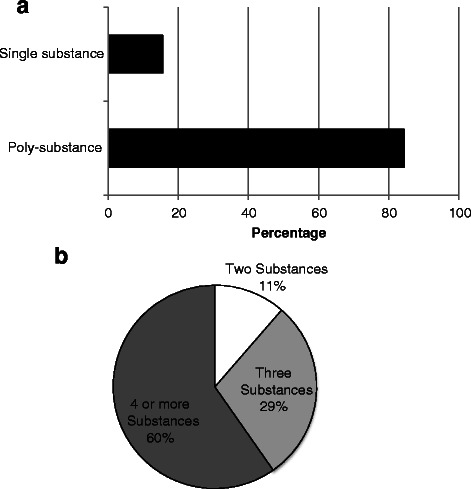
The proportion of the cohort studied that used 2 or more substance was 84.4 % (**a**). A majority of poly-substance group (89 %) used 3 or more substances (**b**)

The data highlights differences in substance use pattern between young patients (19 to 29 years) and those over 30 years old. The proportion of poly-substance users in the cohort studied was over 5 times higher in the younger of the two groups. Figure 1 shows the proportion of the poly-substance users in the study cohort as well as the proportion of users co-using 2, 3 and 4 substances. Specifically, 210 of the 250 patients (84.4 %) in the cohort were poly-substance users and 40 (15.6 %) single substance users. Sixty percent of the poly-substance users used four or more substances. Around 29 % consumed a combination of three substances and 11 % consumed two substances. Nicotine was excluded as a substance of use, since almost all patients (95.6 %) in the cohort were smokers at time of interview, whilst the remaining 4.4 % had stopped smoking.

**Fig. 2:**
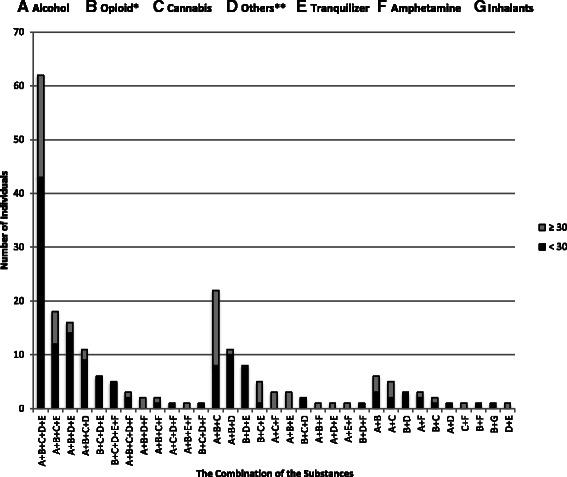
The combinations of the substances consumed by poly-substance users were stratified according to age groups. Opioid users include patients that use pharmaceutical (Tramadol, Codeine) and illicit (Heroin, Morphine) compounds*. The “Others” category refers to the misuse of *Pregabalin, Procyclidine,* and/or *Carisoprodol*

Figure 2 shows the combinations of the substances consumed by poly-substance users. In this group 24.8 % of patients used a combination of alcohol, opioid, cannabis, tranquilisers and one of three prescribed medications. This combination was the most common combination to be consumed especially among those who were less than 30 years of age. This age group was the largest group of the poly-substance users, irrespective of the combination of substances used. Across all the combinations of substances, opioid (used by 80.4 % of poly-substance users) and alcohol (78.4 %) were the most common substances used by patients in the cohort. More young patients used opioid (62.9 %) and alcohol (51.4 %) compared to opioid (29.0 %) and alcohol (31.0 %) use in the over 30 year age group.

**Fig. 3:**
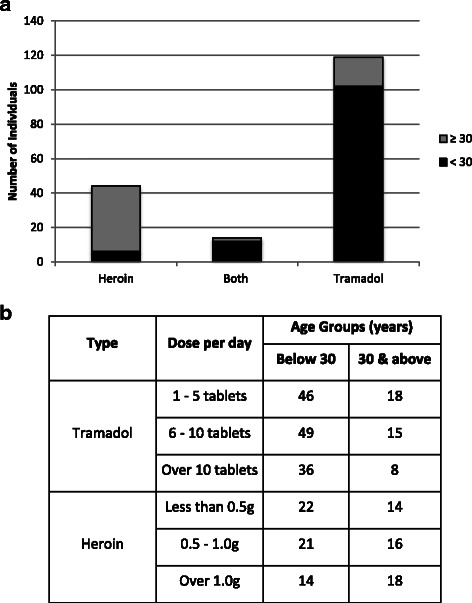
Patients consuming Tramadol and Heroin were grouped according to age. **a** The number of younger patients (<30 years) was over 2 times more than the older patients (≥30 years). The young users tend to use pharmaceutical opioid (57.6 %), in particular Tramadol. In contrast, older patients (>30 years) tend to use illicit opioid (22.6 %), in particular Heroin. **b** The amount consumed of Tramadol and Heroin per day across age groups

Figure 3 shows the breakdown of opioid use, the most common substance used by patients. Classes of opioid consumed included illicit (Heroin, Morphine) and pharmaceutical opioid (Tramadol, Codeine). There were a higher proportion of heroin users among the older group (≥30 years). In contrast, patients below 30 years of age preferred pharmaceutical opioid (57.6 % of Tramadol and 3.4 % of Heroin in under 30 year olds versus 9.6 % of Tramadol and 21.4 % of Heroin in the over 30 year category). Despite this preference, patients in the age group below 30 years used both prescription and illicit opioid excessively in comparison to other age groups. The substantive use of pharmaceutical opioids (an average of 10 tablet or more) was mainly observed in the age group below 30 years (20.9 %) in contrast to the over 30 year group (4.6 %). Over 67.2 % of opioid users reported the use Tramadol on a daily basis with an average of 8 to 9 tablets (of 100 mg/tablet) consumed orally per day. The mean age of initiating use of Tramadol was 20.8 ± 0.5 years with an average duration of use ranging from 5 to 6 years.

**Fig. 4:**
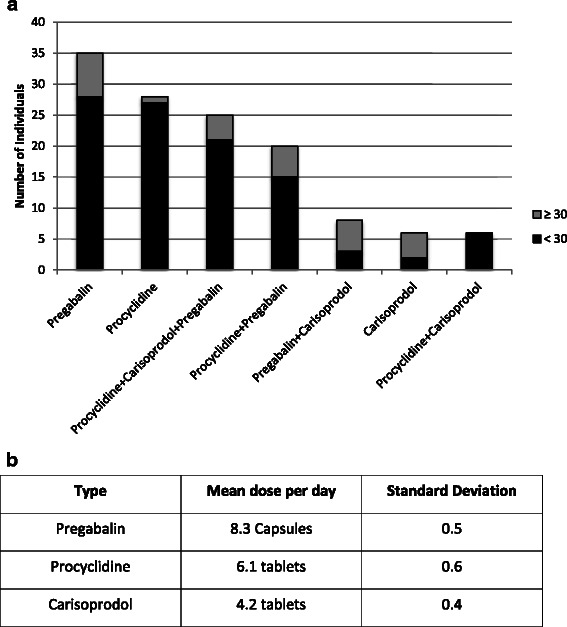
An overview of the misuse of prescription medication. **a** shows that the majority of prescription medication users are in the age group below 30 years old. **b** The average consumption per day of the prescription medication among users

Figure 4 shows the pattern of use of other types of prescribed medications among patients that used poly-substances. Over 60 % of the poly-substance users use prescribed medication such as Pregabalin, Procyclidine and Carisoprodol. Of these 3 specific compounds, they were consumed either as a single compound or as a combination of the 3 compounds. Pregabalin was the most common in this group with over 68 % using this compound (27 % of in single and 41 % in mixture). This compound was consumed at an average ranging from 7 to 14 capsules per day (75 mg and/or 150 mg). Procylidin is the second most common (61 %) with an average of 6 to 7 tablets used per day. The least common was the Carisoprodol (31 %) with an average use of 4 to 5 tablets per day. Alarmingly, these prescription drugs were readily available to the younger cohort with the use of prescribed medications for non-medical use found to be more common in young individuals with the mean age of first use at 20 years old for duration of approximately of 4 years.

## Discussion

### Socio-demographic and clinical characteristic

In this male cohort that represent patients from throughout the UAE, 62.4 % of patients with substance use disorder were in the youngest age group (below 30 years). Other socio-demographic factors that were observed in substance use patients of this cohort were unemployment and single marital status. Although there appears to be a substantial proportion of the patient group that were unemployed (46.4 %) and who had not commenced high school (54.2 %), it was not possible to assess the significance of employment status and education level completed since data from the comparison group was not available. However, the outcomes described here were consistent with findings of a review that examined published studies on substance use disorder in the region [5]. AlMarri and Oeii (2009) [5] reported that substance use was common among unemployed young males with limited education. Elkashef et al. 2013 [11] also reported on the importance of employment status in a retrospective study of NRC patients, showing that 60.3 % were unemployed. Therefore, any approach to minimise the risk associated with the proliferation of this problem has to target the young and unemployed who appear to be the most vulnerable group. In some countries substance use have been associated with the economic problems [15]. However, in this population it is not clear whether unemployment was the cause that initiated the use of substances or whether the use of substance hindered their ability to maintain employment. Consequently, the NRC has initiated a financial support program for regular patients and their families as well as coordinating job opportunities with national and local entities for rehabilitated patients.

Another epidemiological characteristic that appeared to correlate with substance use disorder was marital status (*P-value* < 0.001). Single males were the highest percentage in cohort. The overrepresentation of young single men may be due in part to excess in spare time that youth devote to unproductive activity. Peer pressure especially in the recent social drift in the UAE that predispose younger generation to more stress has also been suggested as a factor [16]. Furthermore, over 50 % of the patients had a family history of substance use disorder (56.8 %). In a review of a series of twin studies and family adoption data by Uhl [17] estimated that genes account for some 40 to 60 % of the overall vulnerability to substance dependency. However, the genes underpinning substance use disorder have yet to be confirmed [18, 19]. The identification of the genes that are involved in the addiction pathway may improve our understanding of the disorder and allow the development of possible prevention programs and improvements to treatment approaches.

There was no correlation between substance use and other clinical conditions requiring prescription medication. Only 29.2 % of the SUD patients studied had co-morbidities such as asthma, psoriasis and allergies that required medication. However, none reported the consumption of the types of prescription medication (Tramadol, Pregabalin, Procyclidin and Carisoprodol) that are discussed in the study.

Most of the patients (95.6 %) were smokers at the time they were interviewed for this study. In contrast, only 19.7 % of the comparison group smoked (*P-value* < 0.001). The high prevalence of smokers in this cohort is consistent with the findings in Bassiony (2013) [20] who reported that dependent patients tended to smoke (48 to 97 %). The proportion of smokers in the general population (2.4 to 52 %) was lower and the 28.9 % from this study is consistent with previous reports. In the Bassiony (2013) [20] study, most of the current-smokers reported smoking at a young age, which was significantly associated with substance use at later stages of their life. It emphasises the importance of prevention strategies that direct quit smoking campaigns pitched at the youth will provide early intervention for smoking. These are already in place at some hospitals in the city of Abu Dhabi as part of campaign to minimise risk factors to cardiovascular diseases in the country. The program has been relatively successful in helping young males between the ages of 15 to 30 years quit smoking.

### The pattern of substances use in the cohort

The general pattern of the substance use shows that proportion of the poly-substance use was higher when compared to single substance users in a cohort of patients studied at the UAE National Rehabilitation Centre (NRC) (Fig. 1). The majority of the poly-substance users consumed more than three substances (89 %) with various combinations of recreational, illicit and prescribed substances across different age groups. There were a number of combinations of substances used (Fig. 2) either with similar or different modes of action on the central nervous system (CNS). The simultaneous use of the substances is presumably driven by availability of a substance in the face of the need to constantly feed the reward pathway.

The most common combination of substances used is alcohol, opioid, cannabis, one of three prescription drugs and tranquilisers. Some combinations of these substances; specifically alcohol and tranquilisers; increases the risk of overdose or death in poly-substance users [21]. Overall, opioid and alcohol were common in most poly-substance combinations and is therefore, not surprisingly, the two common drug groups that are used among most of the cohort participants. Degenhardt et al. (2014) reported that an estimated 1.37 million users were identified in the North Africa and Middle East region in 2010 [22], the third largest group of opioid dependence patients, in terms of absolute numbers. In addition, Elkashef et al. (2013) [11] reported alcohol (41.3 %) and heroin (16.3 %) were the most common substances that were used by patients from the UAE NRC. This pattern is consistent with findings of this study. The work described in this report expands on the ElKashef et al. (2013) [11] study by describing the patterns or combination of substances consumed by UAE patients. Further, it emphasises the use of prescription medication, highlighting the emergence of a new pattern with a shift away from the use of traditional illicit substances. Even though concern regarding the use of prescription for non-medical use is rising, there remains limited epidemiological information relating to the use of prescription medication in the UAE. It is still important to identify and document this rising pattern. As these prescription medications are commonly used for treating certain medical conditions, it is crucial to investigate the risk factors that may predispose an individual to compulsive use of these substances including genetic, developmental or mental co-morbidities [23]. Motivation behind substance use is related to the pattern and outcomes [24, 25] that has previously been classified into two groups; recreational or sensation-seeking and self-medication. In this cohort the majority of the patients reported that they used combination of substances to enhance the effect of the drugs and maintain it for a longer time. In the cohort studied here, the urge to experiment with new substances especially at a young age eventually resulted in a shift toward prescription medication use with the prevailing attitude that these were less hazardous, relative to illicit substances.

The use of cannabis (hashish) was high (65.7 %) among poly-substance group in the cohort studied, however very few individuals developed habitual use of cannabis as the main substance of use. From information disclosed by patients at interview, many perceive cannabis (hashish) as the least detrimental illicit substance and almost all often considered cannabis as the safest option when individuals start experimenting with drugs. As discussed in a review by Copeland and Swift (2009) [26], the debate around the addictive nature of cannabis has been controversial. However, research has identified the mechanism of cannabis receptors that potentially contribute to the tendency to develop cannabis dependency [26]. Even though, it is one of the substances preferred by NRC patients, the move away from cannabis as a primary substance occurs in the early stages of use and may be attributable to a number of factors. As there is a zero tolerance policy toward the consumption of cannabis in the UAE, where possession is punishable with imprisonment for a term of up to 4 years [5], there is an inherent fear of being caught. Further, users have reported the undesirable development of psychotic symptoms experienced with use of cannabis. Finally, there are tendencies to graduate to compulsive use of other illicit substances [27].

The intentional use of prescription medications for non-medical reasons appeared to be increasing and demands the attention of the clinicians and regulators. This is evident by the increasing number of young patients (below 30 years) that present with use of medicines that include tranquilisers and other prescribed medication such as Pregabalin, Procyclidin and Carisoprodol. These drug groups were the preferred substance of use among the age group below 30 years old. Tranquilisers are a common medication with addiction potential that has been used for a longer period of time [23]. It is important that regulators develop uniformity in the legal system when confronted with these newly described trends.

These combinations of substances used reflect the severity of the poly-substance use problem in the UAE. If unchecked, it will create major health concerns by increasing the risk of the drug overdose incidents with the administration of high doses of the same drug classes as well as, fatal adverse reaction that may come with combining multiple drug classes [28]. Diagnostically, poly-substance use disorder is clinically challenging, because the criteria and the scale of diagnosis require further evaluation and research. It is not only important to determine the number of substances used, poly-substance users have been shown to be at higher risk of psychological co-morbidities, and impaired cognitive functioning that interferes with treatment outcomes [2]. Therefore, the detailed description and the pattern of poly-substance use among this cohort was important as it will help in targeting specific substances combinations in order to establish effective diagnostic tools, prevention and treatments strategies.

### Pattern in opioid use (pharmaceutical & illicit)

Opioid is the most common substance of use among patients of this cohort. Tramadol use among opioid users was significantly high (*P-value* <0.0001) when compared to the use of the illicit opioid (Heroin). In this group, 67.2 % use Tramadol compared to the 24.8 % that use heroin. Figure 3 illustrates the break down of the number of individuals in this cohort that use Tramadol, heroin and both of the substances across the two age groups that were studied (below and above 30 years old).

Tramadol is an analgesic agent used to treat acute and chronic pain [29] and range of other conditions [30]. Dependency is a potential risk with more studies reporting incidents of Tramadol overdose that appears as seizures and respiratory depression [31]. In a recent report by Substance Abuse and Mental Health Service Administration (SAMHSA) in the United States, an increase of 47 % observed in emergency departments involved the use of Tramadol with other prescription medication was observed between 2005 and 2011 [32]. From the Tramadol related incidents at these emergency department incidents in 2011, 38 % were admitted to hospitals or transferred to other facility. Among the admitted cases one third (34 %) of the patients were 34 years of age or younger, which is similar with the observations for this cohort. The younger group (below 30 years old) of this cohort appear to be replacing traditionally used opioid (Heroin and Morphine) with synthesised/pharmaceutical analgesics. The trend observed in this study is consistence with United Nation 2014 findings which indicated that the use of opioid analgesic and prescribed medication, in particular Tramadol use in middle eastern countries, had increased significantly over the last decade [6]. There was also an increasing number of patients using pharmaceutical opioid admitted to the NRC as previously reported [11]. The use of the prescribed form of opioid appears to be a new trend among opioid users that requires urgent attention [21]. In this cohort from UAE, Tramadol was preferred compound by young adults as it is easy to administrate and from the patients’ comments at interviews, it appears to be readily accessible most of the time to maintain their dependency. Further, it produces the reinforcing urge; hence high doses were taken frequently, which increases the risk of complications including overdoses and seizures [31]. Yet, there remains little known about the risk factors that leads to using pharmaceutical opioid including Tramadol in vulnerable individuals [23]. Even though, Tramadol is a controlled medication in the UAE that can only be purchased with a prescription, it appears to be readily available to the extent it is a major compound of use seen at the NRC. Methadone use was not observed, as this prescribed form of opioid is not available in the UAE. Future studies will be required to determine risk factors of using or transition to develop pharmaceutical opioid use that will lead to the development of effective public awareness strategies to minimise the risk of use of pharmaceutical opioid [33, 34]. The increasing preference for pharmaceutical compounds over illicit alternatives demands that regulators consider alternative public campaigns targeting vulnerable groups [24, 34], which are the young in the UAE population.

### Trends in other prescribed medication use

There is an alarming trend emerging among poly-substance users of this cohort. Over 60 % of the poly-substance users reported the use of other prescribed medication such as Pregabalin, Procyclidin and Carisoprodol. Pergabalin is gamma-aminobutyric acid (GABA) analogue that is used to treat epilepsy, neuropathic pain and generalised anxiety (GAD). It is efficient in treating other spectrum of diseases such as postherpetic neuralgia and fibromyalgia [35]. Even though, the mechanism of the pregabalin remains unclear, there is a potential risk of non-medical use. Concerns have been reported in various studies [35–37]. Procyclidin is an anticholinergic medication that has been reported commonly used for non-medical purpose [38]. Dooris and Reid (2016) also reported that patients feigned symptoms in emergency departments in order to obtain Procyclidein [39]. Buhrich et al. (2000) reported the common use of the anticholinergic among poly-substance users [40]. In addition, Carisprodol is a skeletal muscle relaxant that can be consumed for non-medical use in excessive dosage [41]. Reeves and Burke (2010) [42] documented in a review paper study involving Carisprodol use and reported an increase in emergency department incidents involving Carisprodol use from 17,366 to 19,513 between 2004 and 2005 in the US. Further, this study reported the recent classification of the Carisprodol as a controlled medication in certain states in the US and restriction applied to the use in European countries, due to the potential risk of dependency.

In this study we have reported the use of these medication (in single or mixture) as a phenomena among poly-substance users of below 30 years old. The availability of these forms of prescription medication as alternatives to fuel a patient’s reward system is providing an array of substitutes to Tramadol. It also allows users to alternate between different types of these prescription medications to maintain their dependency. The use of theses medications as mixture is a major health concern due to the potential risk of toxicity that can lead to overdose and death [6] and action by the local authorities is required. Furthermore, the amount of these medications being consumed is not negligible, and it appears to be the next proliferating challenge, which public health and drug control agencies in the UAE will face.

### Challenges

There were several challenges encountered in this study. The lack of participation of female participants only allows conclusions drawn on male patients. Since 95 % of the patients registered with the NRC are male, it is challenging to recruit female patients. The majority of female patients in the UAE receive treatment for substance use disorder in psychiatric wards in hospitals. Future studies need to be expanded to include different recruitment strategies targeting this group of patients to allow characterisation of patients of both genders with substance use disorder in the UAE.

## Conclusion

The proliferation of the poly-substance use disorder is posing an increasing risk to public health in the UAE. There is an increasing rise in the use of pharmaceutical opioid and prescribed medications, in place of the traditional illicit substances in the UAE population. With only limited information on the potential harm caused by this pattern of substance use, there will be an increase in the demand for data to describe the specific nature of these trends towards improving targeted prevention strategies with effective medical intervention. More emphasis on the prevention and education programs from drug use is required such as proposed a mong West Asian societies using mass media to promote awareness of prevention programs and highlighting the risks posed by both illicit and new emerging substances [20, 43, 44].
